# Spatial variation of zero fruits/vegetables consumption and associated factors among children aged 6–23 months in Ethiopia: geographical weighted regression analysis

**DOI:** 10.3389/fnut.2024.1374845

**Published:** 2024-05-16

**Authors:** Werkneh Melkie Tilahun, Mulat Belay Simegn, Habtamu Geremew, Zenebe Abebe Gebreegziabher, Lamrot Yohannes Abay, Tigabu Kidie Tesfie

**Affiliations:** ^1^Department of Public Health, College of Medicine and Health Sciences, Debre Markos University, Debre Markos, Ethiopia; ^2^College of Health Science, Oda Bultum University, Chiro, Ethiopia; ^3^Department of Epidemiology and Biostatistics, School of Public Health, Debre Birhan University, Debre Birhan, Ethiopia; ^4^Department of Environmental and Occupational Health and Safety, College of Medicine and Health Sciences, Institute of Public Health, University of Gondar, Gondar, Ethiopia; ^5^Department of Epidemiology and Biostatistics, College of Medicine and Health Sciences, Institute of Public Health, University of Gondar, Gondar, Ethiopia

**Keywords:** zero fruits or vegetables consumption, children, spatial analysis, Ethiopia, DHS

## Abstract

**Background:**

After 6 months, nutrient-dense, varied diets containing fruits and vegetables are crucial to supplement breastfeeding. Like many other low-income countries, Ethiopia has very low FV consumption. Zero vegetable or fruit (ZVF) consumption has been shown to significantly raise the risk of non-communicable diseases and has been ranked among the top 10 risk factors for mortality. And it is associated with poor health, an increased risk of obesity, and a higher risk of non-communicable diseases. Thus, this study’s goal was to investigate the spatial distribution of ZVF consumption and its spatial determinants among children aged 6–23 months in Ethiopia.

**Methods:**

A cross-sectional study design was employed. A total of 1,489 weighted samples were included from kids’ datasets from the 2019 Ethiopian mini-demographic and health survey. STATA version 16, ArcGIS version 10.8, Kuldorff’s SaTScan version 9.6, and MGWR version 2.0 software were used for analysis. Spatial regression analyses (geographical weighted regression and ordinary least squares analysis) were conducted. Models were compared using AICc and adjusted *R*^2^. A *p*-value of less than 0.05 was used to declare statistically significant spatial predictors, and the corresponding local coefficients were mapped.

**Results:**

The spatial distribution of ZVF consumption among children aged 6–23 months was non-random in Ethiopia. Spatial scan analysis revealed a total of 120 significant clusters. Maternal education, wealth status, age of the child, place of delivery, number of under-five children in the house, and current pregnancy status were significant predictors of the spatial variation of ZVF consumption.

**Conclusion:**

Significant geographic variation in ZVF consumption was found in this study throughout Ethiopia’s regions. Significant predictors of the spatial variation in ZVF consumption were maternal education, wealth status, child age, place of delivery, number of under-five children in the home, and status as a pregnant woman at the time of birth. Therefore, in order to improve children’s adequate consumption of fruit and vegetables, area-based interventions that can consider these significant factors into account are needed.

## Introduction

A balanced diet should include a variety of fruits and vegetables (FV) since they are excellent providers of dietary fiber, vitamins, and minerals, as well as a number of other helpful non-nutrients like flavonoids, plant sterols, and other antioxidants. Eating them contributes to making sure that several of these vital elements are taken in sufficient amounts (1). It is recommended to have one vegetable serving with every meal, and consuming no FV at all is an unhealthy practice, even if there is no consensus recommendation for the ideal amount of servings of FV per day for children older than 6 months (2). To enhance general health and lower the risk of certain non-communicable diseases (NCDs), the World Health Organization (WHO) recommends consuming approximately 400 grams of FV daily as part of a healthy diet low in fat, sugar, and sodium (1).

After 6 months, children undergo rapid growth and development, for which breastmilk alone is inadequate. Thus, it’s fundamental to supplement breastfeeding with nutrient-dense, diversified diets that include fruits and vegetables (3, 4). Zero vegetable or fruit consumption (ZVF), which is one of many elements of minimum dietary diversity (MDD), is a novel metric introduced by the United Nations Children’s Fund (WHO-UNICEF) to assess infant and young child feeding (IYCF). ZVF measures the proportion of children between the ages of 6 and 23 months who have not consumed any vegetables or fruits in the 24 h prior to the query (3).

It was discovered that 90.22% of Ethiopians engaged in poor child feeding practices (5). Although there was a notable 9% rise in minimum dietary diversity from 2011 to 2016 and a 3.4% reduction in the same period, only 4.1% of Ethiopian children aged 6–23 months were fed the minimum permissible diet (6).

With West and Central Africa having the greatest incidence (56.1%) and Latin America and the Caribbean having the lowest (34.5%), the global prevalence of ZVF consumption was 45.7% (3). Like many other low-income countries, Ethiopia has very low FV consumption (7–10). According to one study, 1.5% of Ethiopians consume FV (10), and 69.3% of children in Ethiopia between the ages of 6 and 23 months consume ZVF (11). According to a study done in Southern Ethiopia, 7.2 and 39.1% of the children consumed FV rich in vitamin A, respectively (12), while a study done using nationally representative data showed that 28.2 and 10.3% of children consumed both vitamin A-rich and other FV (13). In 2019, this prevalence rose to 39% (14).

Zero vegetable or fruit consumption during this crucial developing phase has been shown to significantly raise the risk of non-communicable diseases, and WHO-UNICEF has ranked it among the top 10 risk factors for mortality globally (2, 3). Low fruit and vegetable consumption has a major negative influence on public health; globally, it is responsible for 1.7 million (2.8%) deaths and 16 million (1.0%) disability-adjusted life years (DALYs) (15, 16). Another piece of evidence revealed that up to 2.635 million deaths are thought to occur annually worldwide as a result of poor fruit and vegetable consumption (17). In 2017, it was predicted that 3.9 million deaths globally could be attributed to insufficient consumption of fruits and vegetables (2). A lower intake of fruits and vegetables is associated with poor health, an increased risk of obesity (1), and a higher risk of non-communicable diseases such as hypertriglyceridemia, stomach cancer, colorectal cancer, and cardiovascular disorders (1, 9, 15–19). Furthermore, studies revealed that consuming more fruits and green leafy vegetables is linked to a markedly lower risk of type 2 diabetes (20, 21). Furthermore, over half of young Ethiopian children suffer from anemia and micronutrient deficiencies like vitamin A (8). In all, only 13.3% of children in 2011 and 24% in 2016 attained MDD (22), and the nutrition quality of infants and young children in Ethiopia between the ages of 6 and 23 months was impacted by these incorrect complementary feeding methods (5).

Previous studies conducted in Ethiopia revealed inappropriate child feeding practices (5), poor vitamin A-rich food intake (14), and dietary diversity (22, 23) among children was distributed non-randomly across Ethiopia’s regions. Spatial variation of folate nutrition (24) and crop diversity in agroforestry home gardens (25) was observed between farming systems in Ethiopia. A study conducted using the 2019 Ethiopian mini-demographic and health survey data revealed that factors such as middle and rich household wealth quintile, 25–34 maternal age, Muslim religion, having more than four ANC visits during pregnancy, children in the age group of 12–23 months negatively and being married, and health facility delivery positively associated significantly with children’s ZVF consumption (11).

Despite the fact that variations in crop diversity and availability, non-random distribution of dietary diversity, consumption of foods high in vitamin A, and inappropriate feeding practices for infants are indicated, there is no evidence to determine the spatial distribution of ZVF consumption across Ethiopia’s regions. Investigating the spatial distribution of ZVF consumption across Ethiopian regions is advantageous for targeting undernutrition, ensuring proper child feeding practices, and lowering morbidity and mortality from non-communicable diseases in later life.

## Objective

This study aimed to investigate the spatial distribution of ZVF consumption and its spatial determinants among children aged 6–23 months in Ethiopia.

## Method and materials

### Data sources, setting, population, and sampling design

This study has been conducted based on the 2019 Ethiopian mini-demographic and health survey (EMDHS) dataset, a nationally representative household survey conducted between March 21 and June 28, 2019. The survey was collected across the nine regions and two city administrations that were available at the time of data collection. As part of the sample frame, 149,093 enumeration areas (EAs) were created for the 2019 Ethiopian Population and Housing Census (EPHC). To select participants, a two-stage stratified sampling technique was used. A total of 305 EAs (93 in urban and 212 in rural areas) were chosen in the first stage using a probability proportionate to the size of the EA (based on the 2019 EPHC frame). An equal allocation method was used to choose the sample, and 25 EAs were chosen from eight areas. However, from the three major regions (Amhara, Oromia, and the Southern Nations, Nationalities, and Peoples’ Region (SNNPR)), 35 EAs were chosen. During the second round of choosing, a set number of thirty households were chosen with an equal chance of systematic selection. Interviews were conducted with all females aged 15 to 49 who either lived permanently in the chosen households or were guests who spent the night before the study. Children aged 0 to 59 months had their anthropometric measurements taken in each of the chosen households, and women between the ages of 15 and 49 were questioned (26). Children aged 6–23 months were included. Thus a total of 1,489 weighted sample from Kids Record (KR) file were included. The data were derived from^1^ based on online request and permission.

### Variables of the study

#### Outcome variable

Our dependent variable was ZVF consumption among children aged 6–23 months. Percentage of children aged 6–23 months who did not consume any vegetables or fruits during the previous day. This indicator is based on consumption of vitamin A-rich fruits and vegetables and other fruits and vegetables described in the MDD indicator above. These were part of the 24 h dietary recall used to estimate MDD in the DHS data set. Children were counted if there was no consumption of either food group (15, 27).

#### Independent variables

The explanatory variables included in our study were based on their significance in the previous study conducted on similar data, and such variables were wealth index, maternal age, ANC visits during pregnancy, age of the child, marital status, place of delivery (11), and recommendations from experts (nutritionists and clinical midwives) such as current pregnancy, current breast feeding status, place of residence, maternal education, postnatal care, age at first birth, sex of household head, altitude, and number of under-five children in the house.

### Measurement

The variables considered and the proportion calculated for the analysis were presented in Table 1.

**Table 1:** Measurements of explanatory variables in the study, 2019 EMDHS.

Variable	Measurement
Residence	Proportion of children residing in rural areas
Altitude	Proportion of children residing in altitude ≥2,400 m above sea level
Age of the child	Proportion of children aged 12–23 months
Maternal education	Proportion of mothers who received at least primary level education
Marital status	Proportion of mothers currently married or living with man
Maternal age	Proportion of mothers aged 25 and above
Wealth status	Proportion of children from poor (poorest and poorer) wealth quintile
Sex of household head	Proportion of household with female household head
Pregnancy	Proportion of children whose mother is currently pregnant
Breast feeding	Proportion of children whose mother is currently breast feeding
Age at first birth	Proportion age mothers who gave birth before the age of 18
Place of delivery	Proportion of children who were delivered at home
Postnatal care	Proportion of children who received postnatal care at 2 months
Antenatal care	Proportion of mother who received 4 or more ANC visits during last pregnancy
Number of under-five children	Proportion of children living with more than one under-five children

### Data management and analysis

The MEASURE DHS website provided the data. Excel, Stata version 16, Arc-GIS version 10.7, and MGWR version 2.0 were statistical software that were utilized for data extraction, re-coding, visualization, and other statistical analysis. The study utilized descriptive statistics, and the findings were displayed through the use of text, figures, and tables.

#### Spatial autocorrelation analysis

Global Moran’s I has been used to identify patterns with significant clustering (28). The value is in the interval-1 to 1. If Moran’s I value is zero, values around +1 suggest that the event was randomly distributed and clustered, while values near −1 imply that the event was scattered. Based on statistically significant global Moran’s I (*p* < 0.05) (29), spatial autocorrelation is verified. To determine if the pattern revealed by our data is clustered, distributed, or random, we looked into the spatial pattern of ZVF among Ethiopian children aged 6 to 23 months.

#### Hot spot analysis

Estimating the distribution of events at the local level enables us to examine spatial relations in the study area. Local indicators of spatial autocorrelation, such as the Gettis-Ord Gi*statistic, which identifies statistically significant hot and cold spots by computing a Z score and *p*-value for each grid cell, provide us with more information (30). Usually represented as the ratio of the total values in a given region to the total values, statistical significance is set at a certain confidence level (31).

#### Spatial interpolation

The hot spot analysis was eventually smoothed using an empirical Bayesian Kriging interpolation mapping technique to predict the potential value of un-sampled areas for ZVF among Ethiopian children aged 6–23 months. Because the Bayesian method can reduce the number of false positives and negatives by 50% when compared to conventional methods (31).

#### Spatial scan analysis

Using Kuldorff’s SaTScan version 9.6 software, a Bernoulli-based model spatial scan analysis was carried out to find important primary and secondary clusters of ZVF in Ethiopian children aged 6 to 23 months. In a circular scanning window spanning the Ethiopian region, children who had not consumed any fruit or vegetables over the previous 24 h were classified as cases, whereas those who consumed them were classified as controls. An upper bound was set at a maximum spatial cluster size of less than 50% of the population. Using likelihood ratio tests and matching significance levels based on the default 999 Monte Carlo replications, the most likely clusters were found.

#### Spatial regression analyses

Spatial regression is necessary to comprehend the relationship between the density of specific events and other environmental, demographic, and socioeconomic aspects (32). Therefore, our objective was to understand how other explanatory variables and the prevalence of ZVF consumption among children aged 6–23 months relate to each other.

#### Spatial exploratory regression

This regression evaluates all possible combinations of candidate explanatory variables that best explain the dependent variable within the context of user-specified criteria (33). Thus, possible combinations that can best explain the geographical variation of the outcome variable were first determined. Then, OLS regression was performed and Checks were made on model diagnostics such *R*^2^, adjusted *R*^2^, VIF, Jarque-Bera statistic, joint F, joint Wald, and Koenker (BP) statistic.

The amount of variation in the desired outcome that the explanatory variables in the model could account for was measured using adjusted *R*^2^. The joint F and Wald statistics show the overall model’s significance. If the Koenker statistic is significant (*p* < 0.01), the Wald statistic is a better assessment. The model’s prediction reliability is indicated by the Jarque Bera statistic, which shows that non-random model residuals show a substantial Jarque Bera statistic. The consistency of the spatial processes was evaluated by the Koenker statistic. If it is significant, non-stationarity is the reason. The existence of redundant variables in the model is taken into account by the variance inflation factor (34). Spatial autocorrelation in the residuals was checked. The proved unreliable because residuals were sufficiently autocorrelated. The modeling process was spatially non-stationary or heterogeneous. Consequently, it is essential to use GWR to produce accurate estimates.

#### Geographically weighted regression analysis

For each cluster, the total proportion of ZVF consumption and predictor variables was determined. The GWR utilized dependent and explanatory variables that were similar to those included in the global model. Software called Multiscale Geographically Weighted Regression (MGWR) version 2.0 was used to calibrate the GWR model’s parameter estimates. Weights are determined by a method known as the kernel distance decay function (35). Selecting the kernel type is primarily based on the feature’s spatial configuration. Given that the observations are clustered, we employed adaptive bi-square kernels for geographical weighting, as this type of kernel is suitable for the given data (36).

One crucial factor to consider in GWR is the bandwidth parameter. The number of neighbors or the distance band determines the amount of smoothing that occurs in the model (35). Because it can reduce model complexity due to the number of variables included in the study and the bandwidth as well, the AICc approach automatically identifies a bandwidth with the least AICc (36). Five explanatory factors were included in our analysis, and considerable clustering was noted. We were therefore interested in utilizing the adaptive kernel type found by the golden section search method based on AICc in order to reduce model complexity. The model with the lowest AICc was found to be the best fit for the estimations of local parameters.

The adjusted R2 and AICc values were used to compare the GWR and OLS models. The ideal model was one with a low AICc and a high adjusted *R*^2^ (37). After adjusting for the spatial dependencies found in the OLS model residuals, we lastly tested the spatial autocorrelation of the GWR model’s residuals to see if they were randomly distributed.

### Missing values

Missing data were managed according to the DHS guideline. Thus, the final model was based on complete observation.

### Ethical considerations

Since the study was a secondary data analysis based on publicly accessible DHS records, participant participation and ethical approval were not required. Nevertheless, we asked the MEASURE DHS Program for the data, and we were given permission to download and utilize it.

## Results

Our study included a total of 1,489 weighted samples of children aged 6–23 months. More than two-thirds (68.5%) of children’s mothers were aged 25 and above. More than half (55.45%) of the children’s mothers had attended formal education. Almost all (95.44%) of children’s mothers were married. About 57.98% of children were living in a house with more than one under-five children. Majority (71.34%) were living in rural areas of Ethiopia, while about 36.74% were residing in the Oromia region (Table 2).

**Table 2:** Socio-demographic characteristics of the participants and participant’s mothers, Ethiopia, 2019.

Variables	Category	Weighted frequency (1489)	Percentage (%)
Age of the mother	<25	469	31.50
	≥25	1,020	68.50
Maternal education level	No education	663	44.55
	Educated	826	55.45
Current marital status	Married	1,421	95.44
	Not married	68	4.56
Sex of household head	Male	1,283	86.15
	Female	206	13.85
Wealth index	Poor	613	41.16
	Middle/rich	876	58.84
ANC visits	No	835	56.07
	Yes	654	43.93
Postnatal check up	No	1,293	86.82
	Yes	196	13.18
Residence	Urban	427	28.66
	Rural	1,062	71.34
Region	Tigray	103	6.92
	Afar	21	1.43
	Amhara	323	21.70
	Oromia	560	37.64
	Somali	92	6.14
	Benishangul	17	1.16
	SNNPR	305	20.48
	Gambela	7	0.45
	Harari	4	0.26
	Addis Ababa	48	3.22
	Dire Dawa	9	0.57
Place of delivery	Home	672	45.10
	Health Facility	817	54.90
Age at first birth	<18 years	544	36.53
	≥18 years	945	63.47
Altitude	<2,400	1,249	83.88
	≥2,400	240	16.12
Number of under-five children	One	626	42.02
	More than one	863	57.98
Current pregnancy	No	1,405	94.34
	Yes	84	5.66
Current breast feeding status	No	225	15.11
	Yes	1,264	84.89
Age of the child (months)	6–11	478	32.12
	12–23	1,011	67.88

### Spatial distribution of ZVF

**Figure 1:**
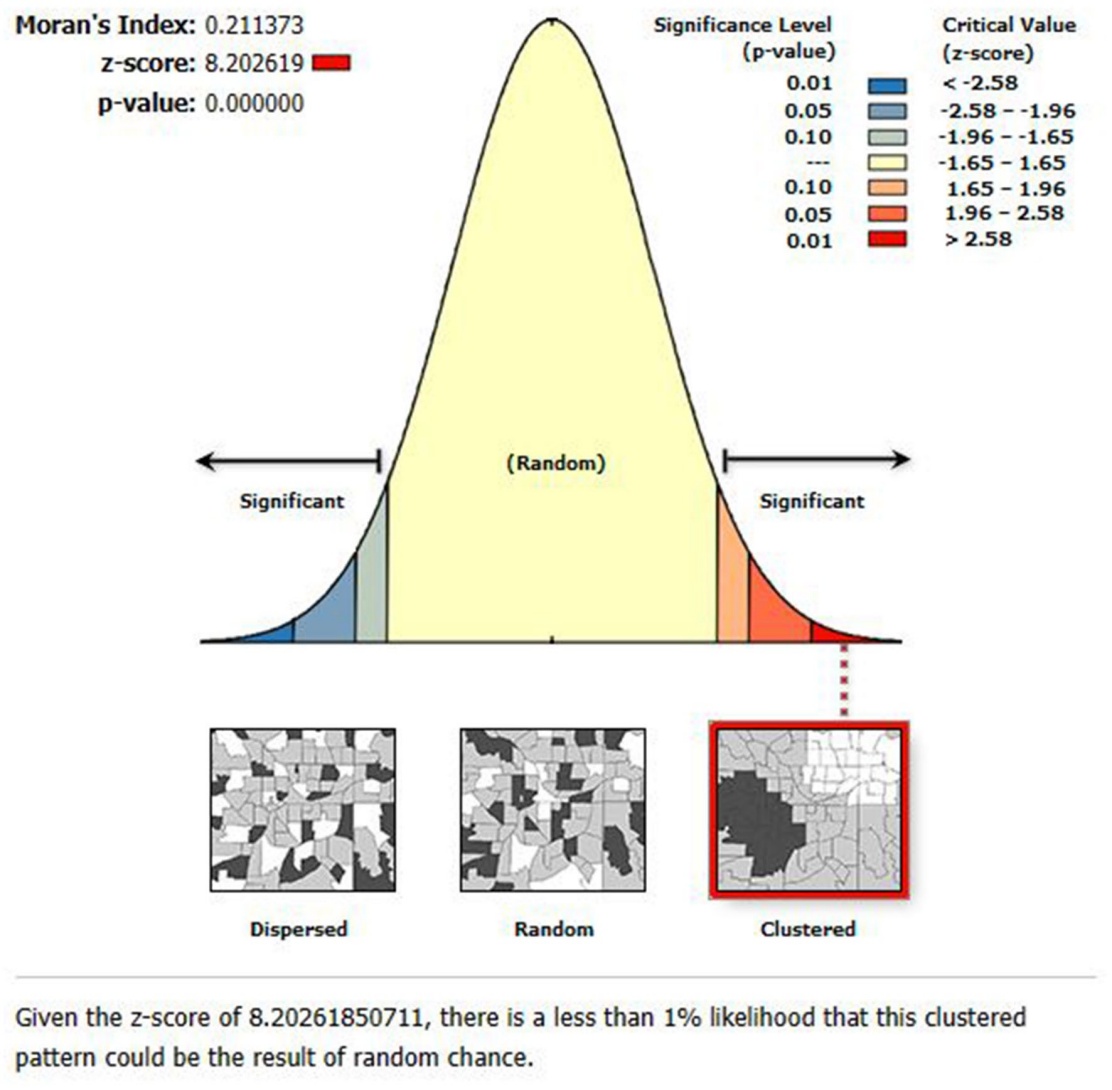
The global spatial autocorrelation analysis of ZVF consumption among children aged 6–23 months in Ethiopia.

**Figure 2:**
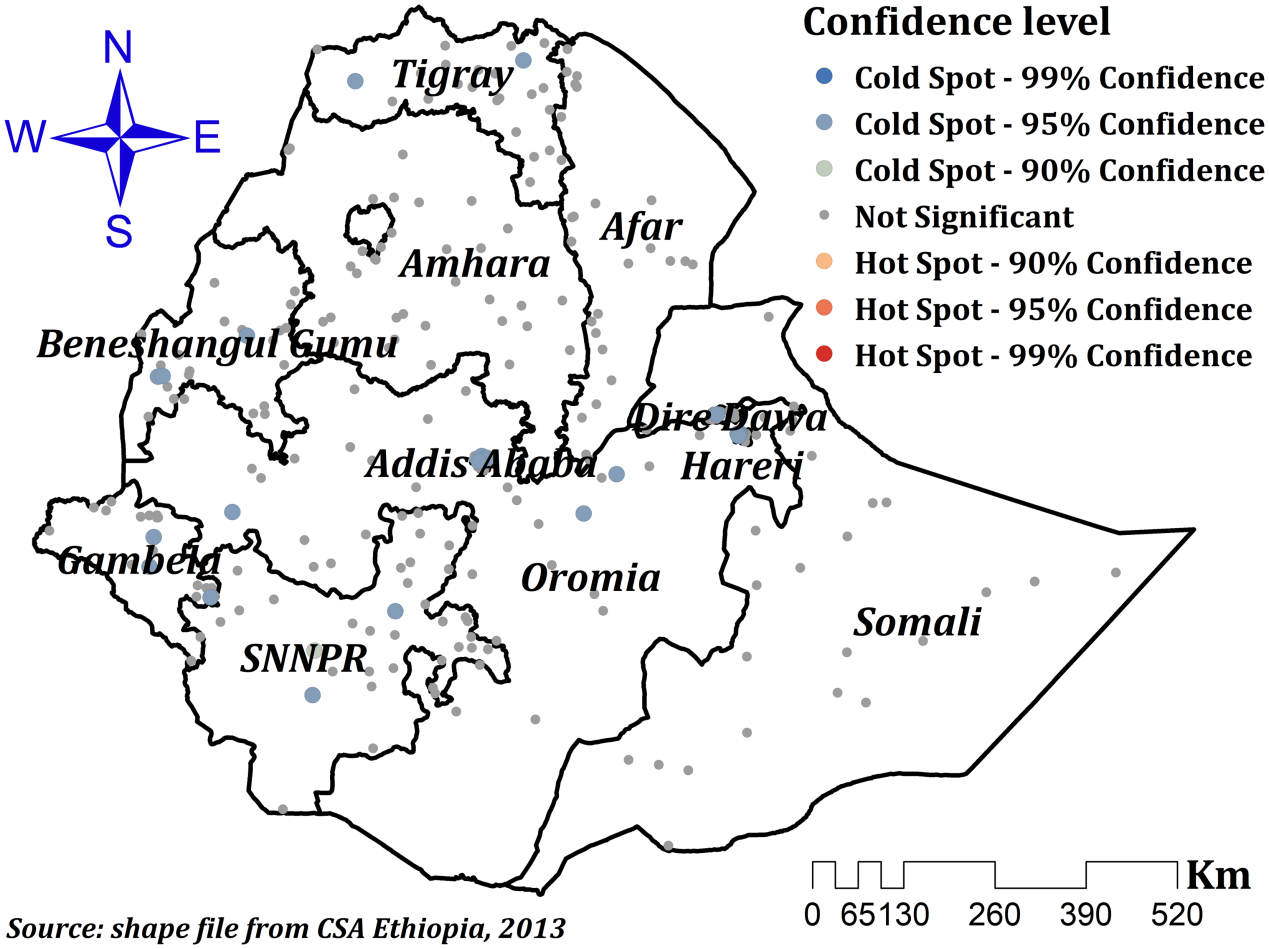
Hot spot analysis of ZVF consumption among children aged 6–23 months in Ethiopia.

Significant spatial clustering of ZVF consumption was observed with a significant global Moran’s index (I) of 0.21 (*p*-value = 0.000) and a Z-Score of 8.20 (Figure 1). The hot spot analysis using the Getis-Ord Gi* statistics showed significant cold spot areas in Benishangul, Gambela, SNNPR, central Oromia, Dire Dawa, Addis Ababa, some parts of Tigray, and some parts of Hareri (Figure 2). This indicates that there was a clustering of low prevalence of ZVF among children aged 6–23 months in the mentioned areas.

### Empirical Bayesian Kriging interpolation

**Figure 3:**
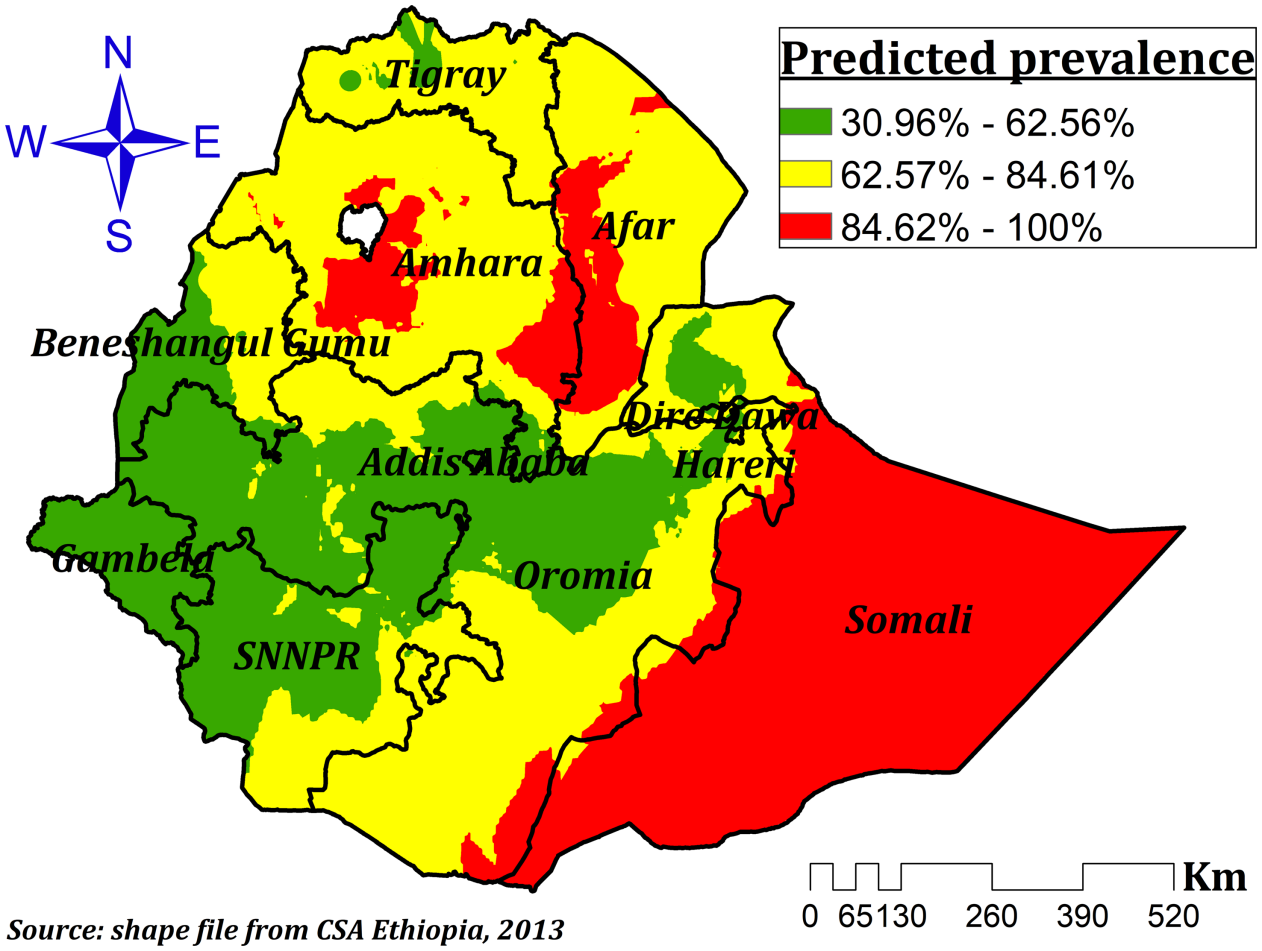
Interpolated prevalence of ZVF consumption among children aged 6–23 months in Ethiopia.

For Ethiopian children aged 6–23 months, the empirical Bayesian Kriging interpolation predicted high and low prevalence areas of ZVF consumption. As a result, ZVF consumption was found to be highly clustered throughout Somali, central and southeast Amhara, and western and north-western Afar, whereas the entire region of Gambela, most of western Oromia, the majority of western Benishangul, the majority of SNNPR, and certain areas of northwest Tigray showed the lowest prevalence (Figure 3).

### Spatial scan analysis

**Figure 4:**
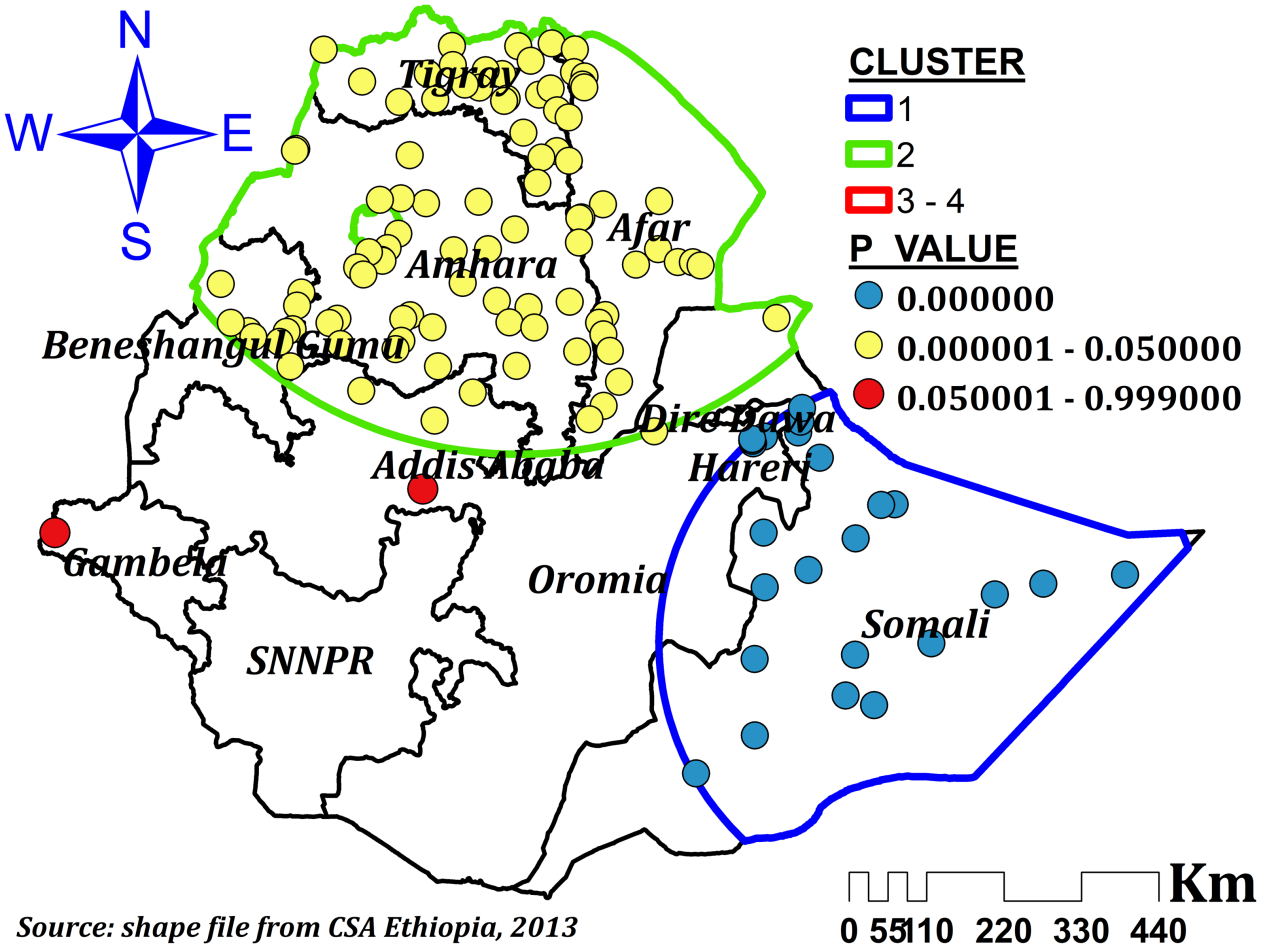
Spatial scan analysis of ZVF consumption among children aged 6–23 months in Ethiopia.

A total of 120 significant clusters were found using spatial scan statistics. Out of those, 96 were secondary clusters and 24 were most likely clusters. The primary clusters were found in Somali, eastern Hareri, and eastern Oromia at 6.639662 N, 44.465855 E/385.91 Km radius (Figure 4). Children residing in these clusters were 46% more likely to have ZVF consumption (RR = 1.46, LLR = 40.46, *p*-value <0.001) compared to children residing outside the window. The second significant clusters were found at 14.344790 N, 39.167347 E/582.62 Km in Tigray, afar, Amhara, parts of northern Oromia, parts of northeast Somali, and the northern half of Benishangul regions (Figure 4). Children from these areas were 28% more likely to have ZVF consumptions (RR = 1.28, LLR = 26.44, *p*-value <0.001) than children outside the window (Table 3).

**Table 3:** Spatial scan analysis results of ZVF consumption among children aged 6–23 months in Ethiopia.

	Cluster type	
	Primary cluster	Secondary cluster
Significant clusters detected	135, 123, 140, 137, 138, 124, 131, 145, 132, 122, 134, 136, 142, 133, 139, 129, 121, 130, 107, 250, 141, 128, 248, 249	10, 16, 11, 15, 12, 17, 3, 14, 39, 2, 13, 35, 7, 6, 1, 37, 27, 25, 38, 23, 36, 8, 9, 24, 20, 19, 22, 18, 56, 21, 78, 45, 83, 29, 46, 62, 82, 4, 61, 44, 84, 34, 58, 57, 59, 60, 55, 85, 30, 33, 54, 81, 74, 65, 64, 63, 31, 75, 53, 51, 26, 66, 47, 32, 48, 70, 71, 76, 49, 68, 72, 50, 67, 79, 52, 165, 73, 80, 162, 77, 100, 43, 163, 148, 40, 126, 119, 166, 159, 42, 99, 158, 164, 161, 160, 127
Coordinates/Radius	6.639662 N, 44.465855 E / 385.91 km	14.344790 N, 39.167347 E / 582.62 km
Population	131	508
Number of cases	129	415
Relative risk	1.46	1.28
Percent cases in area	98.5	81.7
Log likelihood ratio	40.46	82.68
*p*-value	0.000000000000011	0.0000000039

### Exploratory spatial regression

We have used a total of sixteen (16) different significant variables, six from the previous study conducted with similar data (11), and ten other variables in exploratory regression. Accordingly, three possible combinations resulted, and we compared their OLS regression results with the corresponding GWR results. Finally, one combination was found to be a better explanatory and well-designed model. These combinations were proportions of children living with pregnant mothers, educated mothers, children of age 12–23, children delivered at home, and children living with more than one under five children in the house. Thus, our OLS as well as the GWR model considered only these mentioned variables.

### The ordinary least square regression analysis results

A significant combined F and Wald statistic from the OLS model suggests that the model as a whole is significant. The Jarque Bera statistic was significant (*p* = 0.019), indicating that the residuals were not distributed randomly across areas and that the model’s anticipated values were skewed. Breusch-Pagan hetroscedasticity coefficient, as revealed by the Koenker statistic, was statistically significant (*p* = 0.000) (Table 4). It suggests that, as a result of hetroscedasticity or non-stationarity, there is an inconsistent relationship between the proportion of ZVF consumption and other explanatory variables. One of the assumptions of OLS regression analysis was violated when the spatial autocorrelation of the residuals from the OLS model was not independent and was identically distributed (substantially auto-correlated). Consequently, the OLS regression’s findings are not trustworthy. We require GWR to account for the spatial autocorrelation and varying relationship across space in order to map area-specific coefficient estimates of explanatory variables that explain the heterogeneity, improve the reliability of the predictions, and account for the heterogeneous relationship of the process in our model. The model’s explanatory variables’ collinearity or redundancy is measured by the Variance Inflation Factor (VIF). The cutoff value for VIF is generally set at 7.5. In light of this, an explanatory variable having a VIF value higher than 7.5 ought to be removed (34). But all of the explanatory variables in our OLS model had a VIF value of less than 7.5, indicating that multicollinearity was not a significant issue for further investigation (Table 4).

**Table 4:** The OLS regression analysis results for ZVF consumption among children aged 6–23 months in Ethiopia.

Variable	Coefficient	Robust Std. error	Robust probability	VIF
Intercept	0.49	0.073	0.000	–
Proportion of women who were pregnant	0.28	0.11	0.013	1.06
Proportion of women with formal education	−0.38	0.057	0.000	1.55
Proportion of children 12–23 months of age	−0.11	0.067	0.094	1.07
Proportion of children delivered at home	0.15	0.056	0.008	1.65
Proportion of children living with more than one under five children in the house	−0.13	0.060	0.035	1.31

OLS diagnostics		
Diagnostic parameters	Value	*p*-value
Number of observations	296	–
Joint F statistic	23.32	0.000
Joint Wald statistic	137.18	0.000
Koenker (BP) statistic	23.57	0.000
Jarque Bera statistic	7.93	0.019

Model comparison		
Model parameter	OLS (global) model	GWR
*R* ^2^	28.67%	43.4
Adjusted *R*^2^	27.44%	36.80
AICc	71.08	62.25

Std. error, standard error; VIF, variance inflation factor; OLS, ordinary least square; GWR, geographically weighted regression; AICc, corrected akaike information criteria.

### Geographical weighted regression

Similar explanatory variables as those in the OLS model were used for the GWR analysis. The GWR model showed better performance than the global OLS model. In the GWR, the adjusted *R*^2^ increased from 27.44% in the OLS to 36.80%. This suggests that the GWR model explained an additional 9.36% of the variation in the prevalence of ZVF intake across regions. Furthermore, the GWR showed a drop in the AICc value from 71.08 to 62.25 (difference = 8.83) (Table 4). As a result, the geographical heterogeneity was adequately explained by the GWR model.

**Figure 5:**
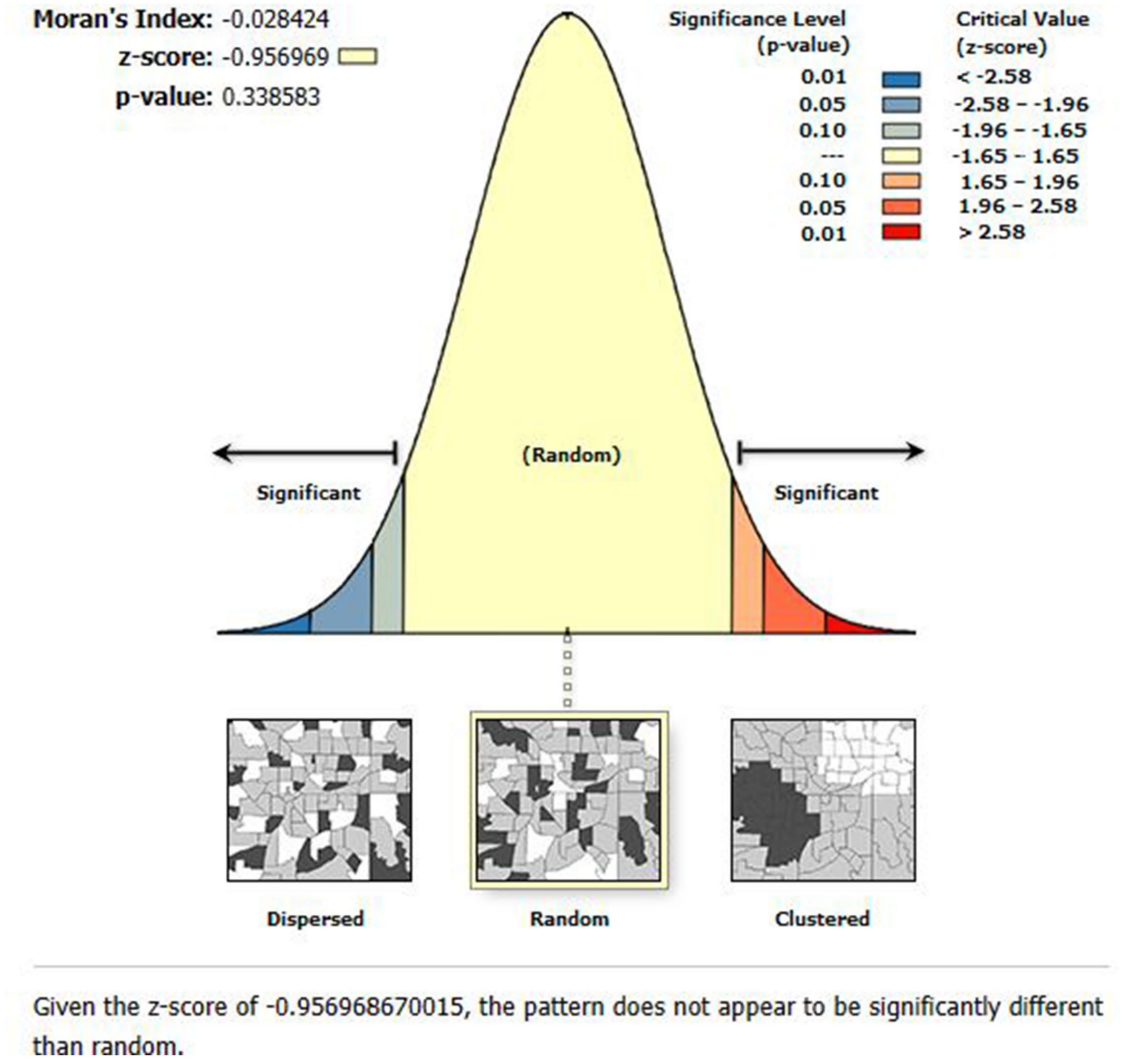
The global spatial autocorrelation analysis of residuals of the GWR model.

We first looked at the residual spatial autocorrelation before interpreting the results of the GWR analysis. Because the residuals’ autocorrelation was randomly distributed following GWR, the residuals’ Moran’s I was-0.028 (*p* = 0.339). The lack of autocorrelation in Figure 5 suggests that the model was appropriately specified and that the variables taken into account in the model adequately explained the spatial dependencies found in the residuals for the global OLS model.

**Figure 6:**
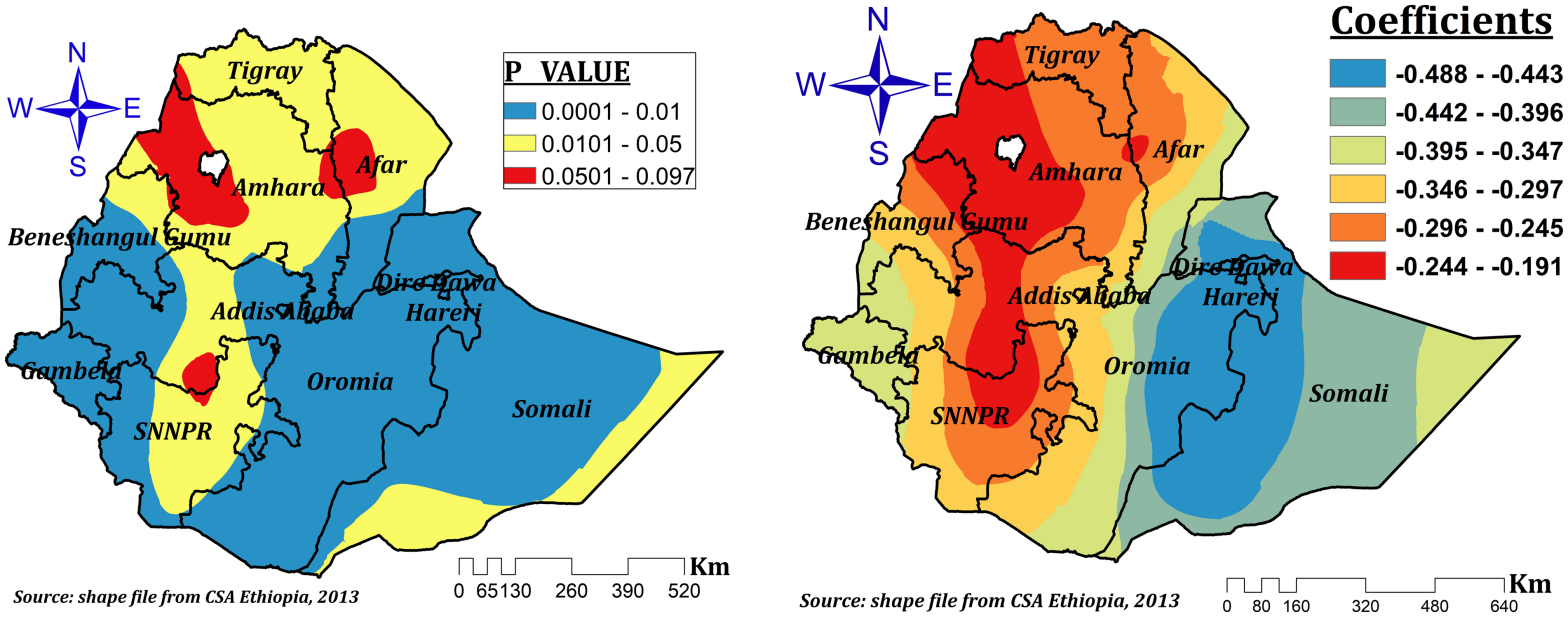
The GWR coefficients and spatially varying significant level of maternal education predicting ZVF intake among children 6–23 months in Ethiopia.

Mother education had a negative effect on children’s ZVF intake in our final model (GWR), which ranged from-0.488 to-0.191 and was significant practically everywhere in Ethiopia. According to Figure 6, a 1% increase in the percentage of women with formal education results in a 19.1 to 48.8% drop in ZVF intake among children. The areas of Dire Dawa, Hareri, eastern Oromia, and southwest Somalia showed the strongest protective effects.

**Figure 7:**
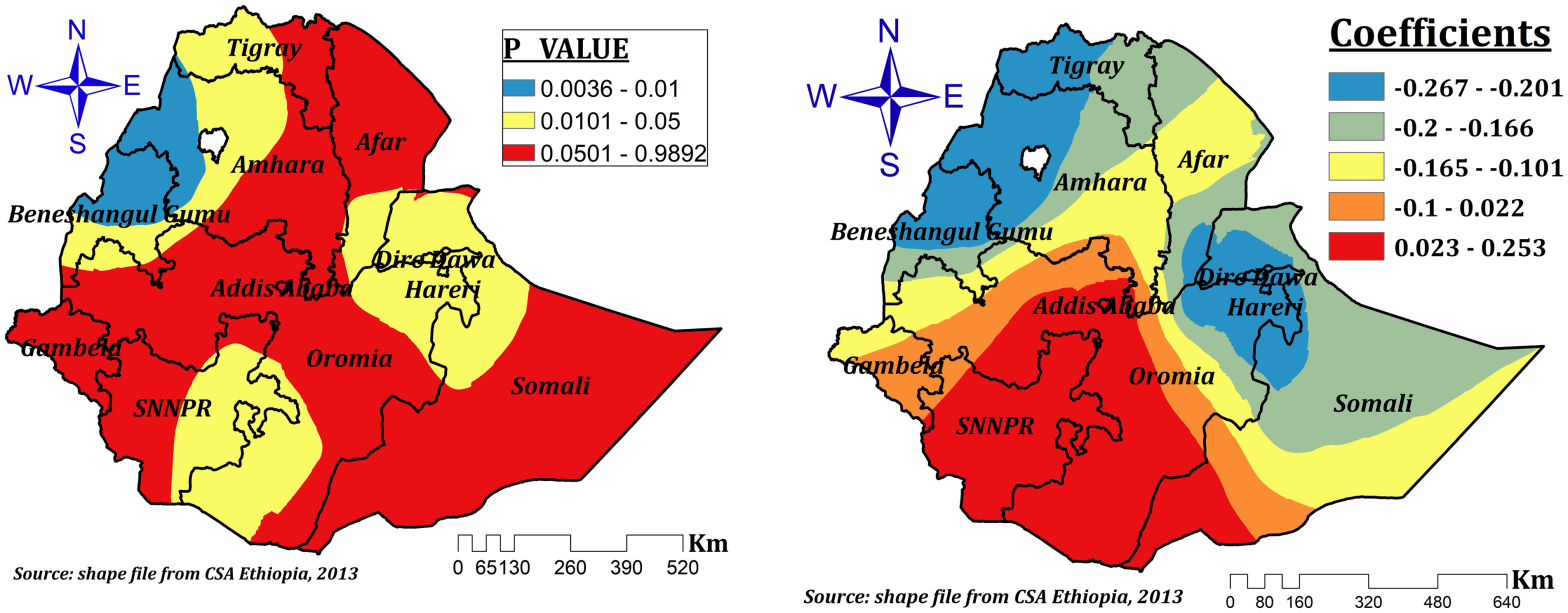
The GWR coefficients and spatially varying significant level of age of the child (12–23) predicting ZVF consumption among children 6–23 months in Ethiopia.

The relationship between the proportion of children aged 12–23 and the prevalence of ZVF consumption was spatially different and both negative and positive, which ranges between-0.267 and 0.253. In the significant areas (northwest Benishangul, west and northwest Amhara, Dire Dawa, Hareri, parts of eastern Somali, some parts of northeastern Oromia, the southern tip of Afar, southeast SNNPR, and the southern tip of Oromia). As the proportion of children aged 12–23 increased by 1%, the prevalence of ZVF intake decreased by 26.7 to 20% in northwest Benishangul, west and northwest Amhara, Dire Dawa, Hareri, parts of eastern Somali, some parts of northeastern Oromia, and the southern tip of Afar regions, while it increased by a range of 2.3 to 25.3% in southeastern SNNPR and the southern tip of Oromia (Figure 7).

**Figure 8:**
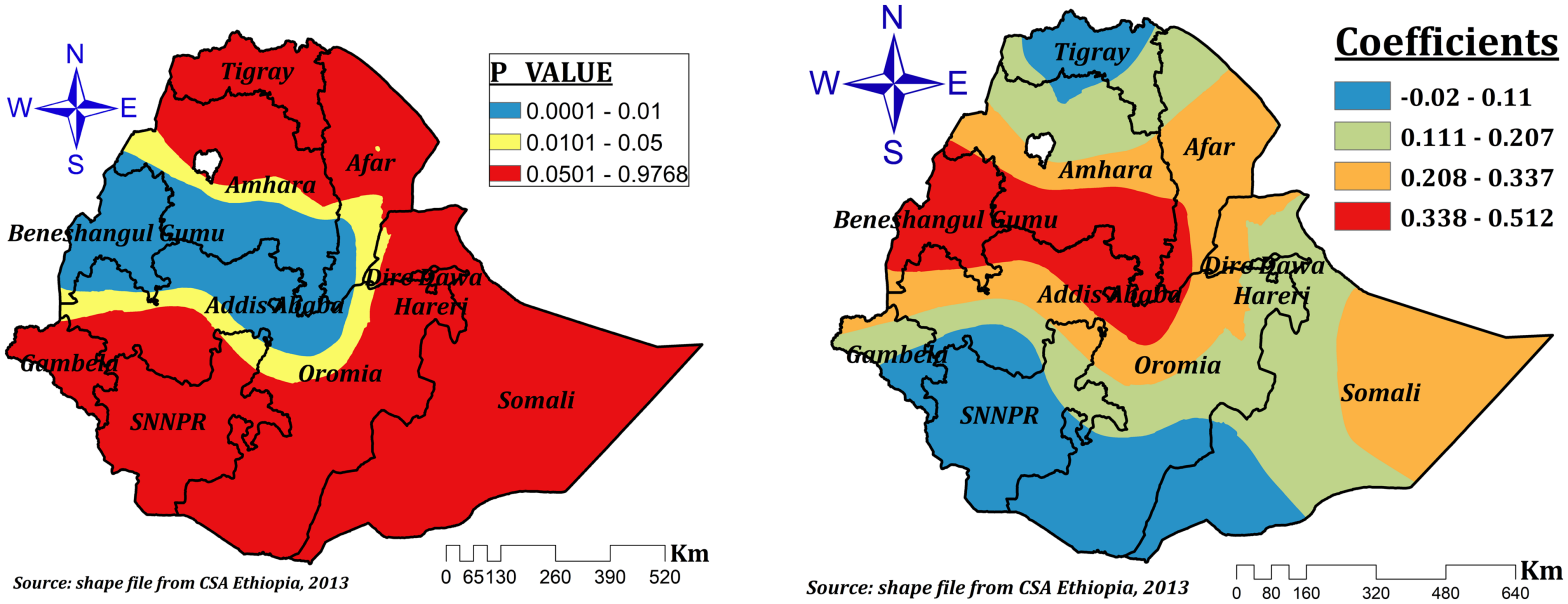
The GWR coefficients and spatially varying significant level of home delivery predicting ZVF intake among children 6–23 months in Ethiopia.

In Ethiopia, the impact of delivery at home on ZVF intake among children aged 6–23 months varies in statistical significance across the regions. The spatial variation of the coefficients, ranging from-0.02 to 0.512, suggests home delivery had both positive and negative spatial effects. Nonetheless, the effect size varies from 0.208 to 0.512 in the significant regions (the entire of Benishangul, the majority of Amhara, southern Afar, some areas of northwest and central Oromia, and Addis Ababa). This suggests that ZVF can rise by a range of 20.8 to 51.2%, with a 1% increase in the percentage of children delivered at home in these areas (Figure 8).

**Figure 9:**
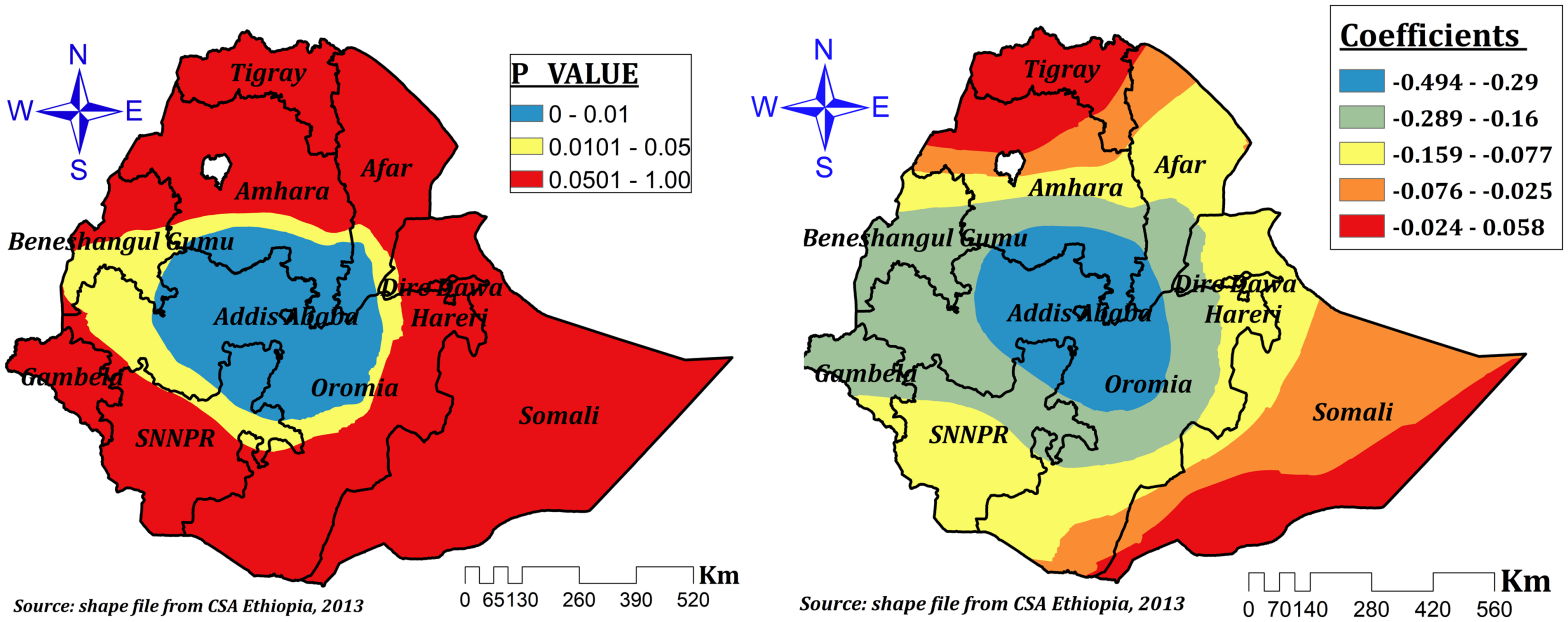
The GWR coefficients and spatially varying significant level of number of under-five children in the house predicting ZVF intake among children 6–23 months in Ethiopia.

The presence of more than one under five children in the house had a varying protective effect in different parts of Ethiopia for ZVF consumption among children aged 6–23 months in Ethiopia. The coefficients vary spatially between-0.494 and-0.024, which indicates that the number of under-five children had a negative spatial effect. However, in the significant areas (Addis Ababa, central Oromia, southern Amhara and afar, some parts of northeast SNNPR, and southeastern Benishangul), the effect size ranges between-0.494 and-0.289. This indicates a 1% increase in the proportion of children living with more than one under five children, which decreases ZVF by a range of 28.9 to 49.4% (Figure 9).

**Figure 10:**
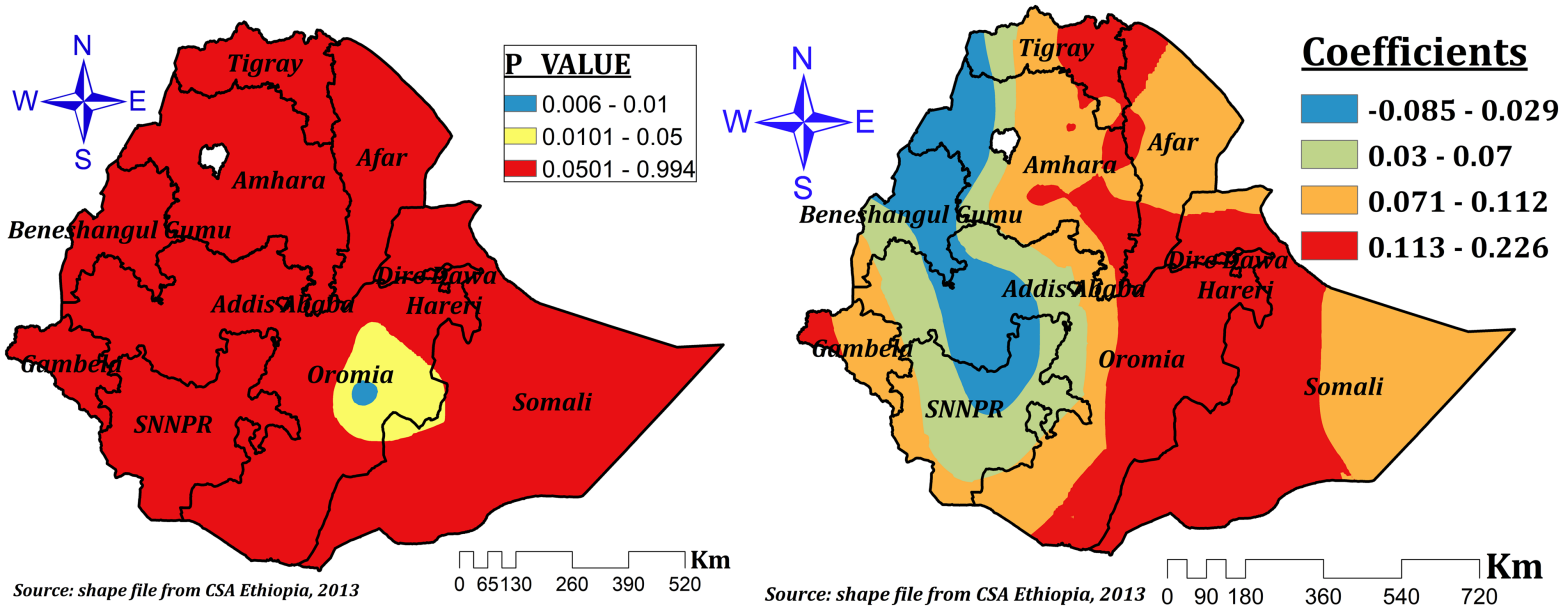
The GWR coefficients and spatially varying significant level of current pregnancy status predicting ZVF intake among children 6–23 months in Ethiopia.

The relationship between mother’s current pregnancy status and the prevalence ZVF was spatially different and both negative and positive which ranges between-0.085 and 0.226. In the significant areas (some parts of southeast and central Oromia and parts of western Somali), a 1% increase in the proportion of pregnant women increases ZVF by a range of 11.2 to 22.6% (Figure 10).

## Discussion

In order to comprehend regional variation and distribution as well as factors contributing to the observed regional variation, this study looked at the spatial variation of ZVF consumption and its associated factors among children aged 6–23 months in Ethiopia. The majority of the Benishangul Gumuz, Gambela, Somali, and SNNPR areas had a highly clustered intake of ZVF. Additionally, a spatial scan analysis resulted in a total of 120 significant clusters. All explanatory factor taken into account in the final model including mother education, the child’s age (12–13 months), home delivery, having more than one child under the age of five living in the house, and being currently pregnant were significant explanatory factors for the regional variation of ZVF. As a result, we mapped these important variables’ local coefficients.

Accordingly, the spatial distribution of ZVF was non-random in Ethiopia and highly clustered in the entire Somali region, central and southeast Amhara, and western and northwest Afar regions. A previous study also indicated that ZVF intake varies spatially (3). And it is also fair to interpret our result relative to other studies conducted to determine the spatial distribution of vitamin A-rich food consumption. Thus, previous studies conducted in Ethiopia revealed that low consumption of foods rich in vitamin A was significantly clustered in the Dire Dawa, Harari, Somali, Afar, eastern Tigray, and southeast Amhara regions (14, 38–40). A study conducted in Vietnam also revealed a spatial variation of vitamin A-rich foods (41). A study conducted in Nigeria also reported geographical differences in the consumption of fruits (42). Fruit and vegetables are one indicator of MDD, and inadequate dietary diversity was highly clustered in the Amhara, Tigray, and Afar regions (22). Moreover, MDD, which involves fruit and vegetable consumption among children (12), is one indicator of appropriate complementary feeding (43). In Ethiopia, inappropriate child feeding practices had a non-random spatial distribution where high clustering was observed in western Tigray, Afar, western Amhara, and Southern Oromia, as well as eastern SNNPR, and low clustering was seen in Addis Ababa, central Oromia, the southern Amhara region, northern SNNPR, southern Benishangul Gumuz, Harari, Dre-Dawa, and the Northern Somali regions (5).

The observed variation in the consumption of fruits and vegetables can be attributed to multiple factors. The type of food consumed and the concentration of micronutrients in Ethiopia can be greatly influenced by a variety of factors, including variations in farming systems across the regions, affordability, utilization, food preferences, lack of nutrition education and storage facilities, and differences in residential food availability (24, 44–46), as well as by the seasonality of fresh vegetables and fruits that are available (3). In addition, prices are rapidly increasing, both in real terms and when compared to cereal. At the end of 2019, vegetables and fruits were significantly more expensive than 15 years earlier. Especially green leafy vegetables show a significant price rise. There is also a significant spatial price variation in Ethiopia, where vegetable prices are 60 percent more expensive in lowland regions than in the Amhara region, where vegetables are the cheapest. Fruit prices in the lowlands are double the prices in the major producing area, the Southern Nations, Nationalities, and Peoples’ (SNNP) region (47). That is the reason for the lowest prevalence, which was seen in the entire Gambela, the majority of western Oromia, western Benishangul, the majority of SNNPR, and some parts of the northwest Tigray regions, where fruit production is comparatively better as spatial and temporal variation in crop diversity in Ethiopia is known (25). Due to rainfall, temperature, and altitude differences in the country, the eastern part of the country (East Hararghe) with vegetables dominating; Central Oromia Regional State (which produces both fruits and vegetables); SNNPR (Gamo Goffa, Woliata and Sidama zones); and eastern Ethiopia (Dire Dawa and Harari) are also well-known production and supply areas of both FV; some parts of Tigray and Amhara regions are the major fruit and vegetable growing areas of the country (48). Other sociodemographic factor variation across the regions may contribute to the spatial variation of ZVF consumption in Ethiopia (4, 49).

Our study revealed that mother education was a spatially significant predictor variable for ZVF consumption. It had a significant negative effect almost across all regions that ranged between-0.488 and-0.191. This indicates a 1% increase in the proportion of women with formal education, which decreases ZVF intake by a range of 19.1 to 48.8% among children aged 6–23 months. This finding is supported by previous reports (3, 18). Other studies also revealed that mother education was positively associated with different micronutrient rich food intakes (14, 40), dietary diversity (22, 23), and the minimum acceptable diet intake (6). The highest effect was seen in less developed regions and this might be due to regional inequalities in educational achievement and opportunities among women (49). Our finding is in contrast to the previous multilevel analysis (11). This might be due to the effect of space and spatial relationships in our analysis (50).

Children’s age (12–23 months) was another significant spatial predictor for ZVF intake. It had a spatially negative and positive effect that ranged between-0.267 and 0.253. A 1% increase in the proportion of children aged 12–23 decreases the prevalence of ZVF intake by 26.7 to 20% in northwest Benishangul, western and northwest Amhara, Dire Dawa, Hareri, some parts of eastern Somali, some parts of northeastern Oromia, and the southern tip of the Afar regions. This finding is consistent with the results of previous studies (3, 11). However, our result also indicated that a 1% increase in the proportion of children aged 12–23 increases ZVF intake by a range of 2.3 to 25.3% in southeastern SNNPR and southern Oromia. This might be due to the lack of availability of fruit and vegetables in these specific areas (48) or decreased maternal care and attention given to children as they grew up (51).

The effect of home delivery had varying statistical significance for ZVF consumption among children aged 6–23 months in Ethiopia. It’s the coefficients that vary spatially between-0.02 and 0.512. However, in the significant areas (entire Benishangul, the majority of Amhara, southern Afar, some parts of northwest and central Oromia, and Addis Ababa), the effect size ranges between 0.208 and 0.512. This indicates that a 1% increase in the proportion of children delivered at home in these areas can increase ZVF intake by a range of 20.8 to 51.2%. Another study also supports this evidence (52). This might be due to the reduced effect of home delivery on good infant and young child feeding practice (53–55), since health facility delivery creates opportunities for advice and encouragement that foster child feeding practice. However, our finding is in contrast to the previous report (11). This might be the reason why spatial analysis is more advantageous over other statistical analysis methods since it integrates space and spatial relationships (50).

The presence of more than one under five children in the house had a varying negative effect in different parts of Ethiopia on ZVF intake among children aged 6–23 months in Ethiopia. It’s the coefficients that vary spatially between-0.494 and-0.024. However, in the most significant areas, the effect size ranges between-0.494 and-0.289. This indicates a 1% increase in the proportion of children living with more than one under five children in the house, which decreases ZVF intake by a range of 28.9 to 49.4%. A previous study revealed that having three or more under-five children in the house increases undernutrition status (56). However, this significant effect was seen in Addis Ababa, central Oromia, southern Amhara and afar, some parts of northeast SNNPR, and southeastern Benishangul, where a better availability of fruits and vegetables is indicated (48). Thus, if the number of under-five children in the house increases, mothers may give better attention to caring for their children.

The relationship between mother’s current pregnancy status and the prevalence ZVF intake was spatially different and both negative and positive which ranges between-0.085 and 0.226. In the significant areas, a 1% increase in the proportion of pregnant women increases ZVF consumption by a range of 11.2 to 22.6%. Mothers may worry more about their pregnancies than their children, which could be the cause of the positive effect of pregnancy on ZVF consumption.

### Strength and limitations of the study

The data used in this investigation were gathered using a validated instrument and are nationally representative. In order to determine hotspots and cold spots with ZVF consumption as well as spatial predictors for this spatial variation in a particular geographic area, we used a spatial analysis. To protect respondent anonymity, our study’s GPS position is, however, randomly moved. Consequently, positional errors amount to 0–2 kilometers in urban clusters, 0–5 kilometers in rural clusters, and 0–10 kilometers in 1% of the rural clusters. This could have an impact on local estimates and make it more difficult to determine where the instances are located.

## Conclusion

Significant geographic variation in ZVF consumption was found in this study throughout Ethiopia’s regions. Significant explanatory factors for the spatial variation in ZVF consumption among children aged 6–23 months were the formal education level of the mother, wealth status, the child’s age, place of delivery, number of under-five children living in the home, and pregnancy status of the woman. Our research shows that improving children’s consumption of fruits and vegetables in hotspot areas is a critical requirement. Therefore, in order to lower children’s rising ZVF consumption rate, boosting household wealth status and expanding access to education should take precedence. Consequently, rather than implementing services randomly in hotspot regions, area-based interventional strategies that take spatial explanatory factors into account would make regional nutritional and educational interventions more cost-effective. Lastly, our findings should encourage public health authorities to develop targeted nutritional education.

## Data availability statement

Publicly available datasets were analyzed in this study. This data can be found here: http://www.dhsprogram.com.

## Ethics statement

Ethical approval was not required for the studies involving humans because this study was conducted using the 2019 Ethiopian demographic and health survey datasets. To use the datasets we granted a permission from the MEASURES DHS program and the authorization letter is attached with files. The studies were conducted in accordance with the local legislation and institutional requirements. Written informed consent for participation was not required from the participants or the participants’ legal guardians/next of kin in accordance with the national legislation and institutional requirements because this study is a secondary data analysis. Thus, it is not applicable.

## Author contributions

WT: Conceptualization, Data curation, Formal analysis, Investigation, Methodology, Project administration, Resources, Software, Supervision, Validation, Visualization, Writing – original draft, Writing – review & editing. MS: Investigation, Methodology, Project administration, Resources, Supervision, Validation, Visualization, Writing – review & editing. HG: Investigation, Methodology, Project administration, Resources, Supervision, Validation, Visualization, Writing – review & editing. ZG: Investigation, Methodology, Project administration, Resources, Supervision, Validation, Visualization, Writing – review & editing. LA: Investigation, Methodology, Project administration, Resources, Supervision, Validation, Visualization, Writing – review & editing. TT: Investigation, Methodology, Project administration, Resources, Supervision, Validation, Visualization, Writing – review & editing.

## Footnotes

^1^ http://www.dhsprogram.com
